# Improving spatial working memory in blind and sighted youngsters using programmable tactile displays

**DOI:** 10.1177/2050312118820028

**Published:** 2018-12-18

**Authors:** Fabrizio Leo, Carla Tinti, Silvia Chiesa, Roberta Cavaglià, Susanna Schmidt, Elena Cocchi, Luca Brayda

**Affiliations:** 1 Robotics, Brain and Cognitive Sciences department, Center for Human Technologies, Istituto Italiano di Tecnologia, Genoa, Italy; 2 Dipartimento di Psicologia, Università degli Studi di Torino, Turin, Italy; 3 Istituto David Chiossone per Ciechi e Ipovedenti Onlus, Genoa, Italy

**Keywords:** Blindness, spatial working memory, training, haptics, tactile displays

## Abstract

**Objective:**

To investigate whether training with tactile matrices displayed with a programmable tactile display improves recalling performance of spatial images in blind, low-vision and sighted youngsters. To code and understand the behavioral underpinnings of learning two-dimensional tactile dispositions, in terms of spontaneous exploration strategies.

**Methods:**

Three groups of blind, low-vision and sighted youngsters between 6 and 18 years old performed four training sessions with a weekly schedule in which they were asked to memorize single or double spatial layouts, featured as two-dimensional matrices.

**Results:**

Results showed that all groups of participants significantly improved their recall performance compared to the first session baseline in the single-matrix task. No statistical difference in performance between groups emerged in this task. Instead, the learning effect in visually impaired participants is reduced in the double-matrix task, whereas it is still robust in blindfolded sighted controls. We also coded tactile exploration strategies in both tasks and their correlation with performance. Sighted youngsters, in particular, favored a proprioceptive exploration strategy. Finally, performance in the double-matrix task negatively correlated with using one hand and positively correlated with a proprioceptive strategy.

**Conclusion:**

The results of our study indicate that blind persons do not easily process two separate spatial layouts. However, rehabilitation programs promoting bi-manual and proprioceptive approaches to tactile exploration might help improve spatial abilities. Finally, programmable tactile displays are an effective way to make spatial and graphical configurations accessible to visually impaired youngsters and they can be profitably exploited in rehabilitation.

## Introduction

Investigating how spatial working memory (SWM) operates in blind and visually impaired people has been particularly attractive, considering it allows one to understand the role of vision in this cognitive system. Several studies have evaluated different spatial abilities in blind people, such as building spatial representations or mental imagery.^1–3^ Nevertheless, it is difficult to convey a general conclusion about how spatial cognitive abilities of blind as compared to sighted people differ, since past research has shown significant performance variability as a function of the degree of visual disability and of the specific ability investigated, such as mental rotation,^4–6^ the building of egocentric and allocentric representations of small or large scale spaces,^7–10^ or spatial auditory recalibration.^11^

As for specific performance in SWM, some studies evidenced similar performance in blind and sighted people, especially when the tasks required passive storage in working memory,^12^ such as when information is stored and recalled as presented without mental modification. It has also been shown that people who are blind perform worse than sighted people in more complex tasks that involve transformation, manipulation and integration of spatial information,^13^ or when they must memorize large amounts of data.^14^ In particular, Vecchi et al.^15^ evidenced that blind people have difficulty memorizing multiple patterns of information, such as memorizing the location of tactually explored targets in two different matrices; on the contrary, no differences between blind and blindfolded sighted participants emerged when they have to integrate the location of targets of two matrices into one matrix. According to the authors, these differences might be due to the difficulty of people who are blind when comes to simultaneously maintaining separated spatial information. Importantly, Vecchi et al.^15^ showed that starting from a tactile exploration, both blind and sighted can retain and process information that is spatial in nature.

Other studies using tactile tasks to evaluate spatial abilities showed that blind people’s performance is superior to the one observed in low-vision and sighted people, suggesting that touch compensates for the absence of sight by allowing for the coding of spatial patterns to emanate from tactile instead of visual input.^16–18^ This observation is in line with studies that show brain activations in visuo-spatial pathways both in blind and sighted persons when acquiring tactile maps,^19^ indicating that cortical recruitment linked to spatial content only minimally depends on the visual system.^20^ Interestingly, blind and sighted people exhibit comparable brain activation^21^ despite having different temporal patterns,^22^ corroborating the existence of a supramodal representation system of spatial layouts.^23^

Overall, the cited studies suggest that touch is one of the most important senses necessary to compensate for the absence of vision in spatial tasks and empirical findings showed that tactile training can enhance acquisition of spatial knowledge.^24^ In particular, Leo et al.^24^ showed the benefits of training for enhancing the spatial abilities of blind and low-vision youngsters by using a Corsi-like spatial memory task implemented with a programmable tactile display. An additional piece of research looked not only at performance, but at *how* touch enables one to acquire spatial information, and how using a particular strategy could affect performance.^25–27^ Indeed, the method of acquiring information can be correlated with the quality of its subsequent mental representation. In fact, exploration modalities influence the quality of the building of spatial representations,^28–30^ and there are differences between blind and sighted persons in haptic strategies.^31^ For instance, studies on exploration strategies show that sighted people spontaneously are more likely to use a single finger of one hand,^31,32^ while blind people employ many fingers and both hands.^33,34^ There seems to be no systematic classification of how persons with different degrees of visual impairment explore unknown tactile objects, even if preliminary studies exist with specific tactile devices.^35^ Finding a possible “ideal” exploration strategy could be useful as a guideline for rehabilitating practitioners.

In the present study, by using the same programmable tactile display as in the study of Leo et al.,^24^ we implemented a mixed within–between design. In particular, we investigated whether, with four weekly scheduled sessions of SWM training, blind and low-vision children and adolescents can improve their performance in both simple and complex spatial tasks. In more detail, in the simple task, participants must retain and reproduce the position of targets displayed on one single matrix (single-matrix), while in the complex task, they have to simultaneously retain and reproduce the positions of targets displayed in two matrices (double-matrix). The results concerning the performance of the blind and low-vision youngsters in the single-matrix task were already published in Leo et al.^24^ and showed that both groups were able to improve their recalling performance across sessions. Here, in contrast to Leo et al.,^24^ we wanted to investigate whether the performance of the blind and low-vision youngsters can improve also in more complex memory tasks. In addition, we also compared the performance of visually impaired youngsters with those of blindfolded sighted participants matched by gender and age. Finally, we investigated the possible influence of exploration strategies on performance in spatial memory tasks. To sum up, we wanted to answer the following research questions:

1. Does the recalling performance improve in complex as well as in simple spatial tasks?
2. Is the degree of visual ability modulating the recalling performance and/or the exploration strategies?
3. Is it possible to identify an ideal strategy which can be exploited in rehabilitation programs?

## Methods

The study entailed a 3 × 4 × 2 mixed design with group (blind vs low-vision vs sighted) as between-subject factors, and number of training sessions (four levels) and type of spatial task (simple vs complex) as within-subject factors.

## Participants

Three groups of children and adolescents took part in the study: a group of blind (BLI; *n* = 8), a group of severe low-vision (LOW; *n* = 8) and a sighted control group (SIG; *n* = 16). Following the World Health Organization (WHO) guidelines, we defined blindness as vision in a person’s best eye with correction of less than 20/500 or a visual field of less than 10°. We define severe low-vision as vision comprised of between 20/200 and 20/400 in the better eye after correction. BLI age ranged from 8 to 18 years (mean age = 11.3; *SD* = 3.4). LOW age ranged from 6 to 14 years (mean age = 11.7; *SD* = 2.9). SIG age ranged from 6 to 17 years (mean age = 11.9; *SD* = 2.9). Participants had no conditions affecting tactile perception, nor did any have cognitive impairment. All the characteristics of the participants are detailed in Table 1. Visually impaired participants were selected by the Istituto David Chiossone in Genoa, which also hosted their testing. The University of Turin recruited and tested the SIG. Before the study began, written informed consent in compliance with the Declaration of Helsinki was obtained either from the legally authorized representatives of the minor participants or from the participant himself of herself in cases of age of majority. The experimental protocol was approved by the local Ethics Committees.

**Table 1.** Characteristics of the participants.

Participant	Gender	Age (y)	Etiology of visual impairment	Age at onset of visual impairment	Residual vision	Braille reader
BLI						
01	F	9	Retinopathy of prematurity	Birth	None	Yes
02	F	13	Congenital cataract	Birth	None	Yes
03	F	16	Retinopathy of prematurity	Birth	None	Yes
04	F	11	Retinopathy of prematurity	Birth	None	Yes
05	M	12	Amaurosis	2 years	Sense of light	No
06	M	8	Retinopathy of prematurity	Birth	None	Yes
07	F	10	Retinopathy of prematurity	Birth	None	Yes
08	F	18	Retinitis pigmentosa (Alstrom Syndrome)	5 years	None	Yes
LOW						
09	M	6	Albinism	Birth	1/10	No
10	F	14	Arachnoid cyst	11 years	1/50	No
11	M	14	Gliomatosis cerebri	1 year	1/25	No
12	F	9	Microphthalmia	Birth	2/12	No
13	M	12	Albinism	Birth	3/20	No
14	F	13	Stargardt disease	Birth	1/10	No
15	F	12	Homocystinuria	Birth	1/20	No
16	F	14	Nystagmus	Birth	1/10	No
SIG						
1	M	6				No
2	F	10				No
3	M	14				No
4	F	14				No
5	M	8				No
6	F	9				No
7	F	13				No
8	F	9				No
9	F	14				No
10	F	17				No
11	M	12				No
12	F	13				No
13	F	11				No
14	F	12				No
15	M	12				No
16	F	16				No

BLI: group of blind; LOW: group of severe low-vision; SIG: sighted control group.

## Materials and procedure

**Figure 1.**
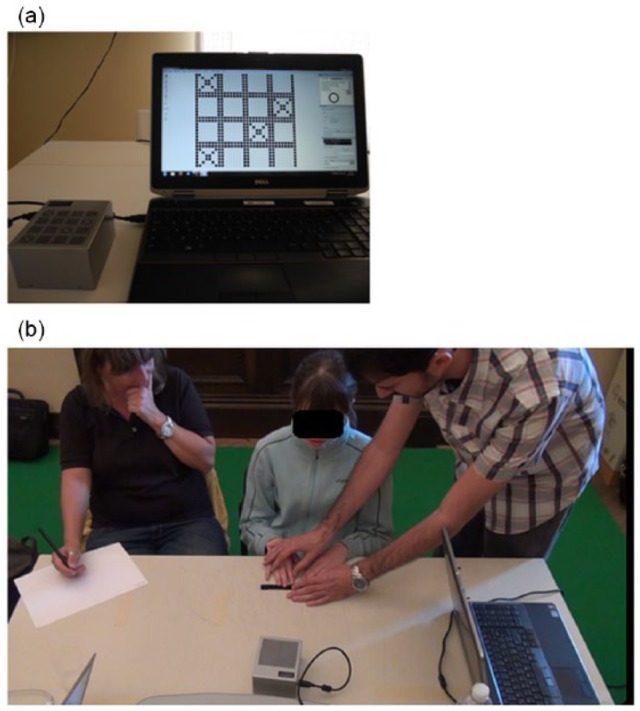
Experimental setup: (a) Experimental setup with the Hyperbraille display on the left side and the PC running the PadDraw software on the right side. The picture shows an example trial of the spatial memory task. (b) An experimenter and a rehabilitation practitioner giving instructions to a blind child. A rehabilitation practitioner was always present during the tests involving visually impaired youngsters. The child’s parents gave informed and written consent for the publication of this figure.

The tasks were performed using Hyperbraille, which is a Pin-Matrix multi-line Braille electronic display that was provided by Metec AG (see Figure 1). The Hyperbraille is composed of an array of 30 × 32 moving pins and it has a screen refresh of 5 Hz. The spacing between adjacent Braille dots was 2.5 mm and each pin raises at about 0.7 mm. The device was connected via USB cable to a standard laptop and controlled by the software PadDraw, MATLAB R2014 and Psychtoolbox 3.0.11.^36,37^ PadDraw is a software developed by Geomobile GmbH within the scope of the FP7 EU Blindpad project.^38^

The three groups of youngsters performed a four-sessions weekly scheduled training. Sighted and low-vision participants were blindfolded so as to remove any visual input. In each session, participants performed two tasks using the programmable tactile display. Before starting the tasks, participants familiarized themselves with the tactile display. For each participant, we adjusted the level of difficulty of the tasks at the beginning of session I of the training according to his or her ability. In particular, the criterion was to find a level of difficulty in which the tasks were neither too easy nor too difficult, while preserving the possibility of observing learning effects across sessions. We did this by aiming at reaching a performance target level of 70% of accuracy, which represented the session I baseline. Once we determined the appropriate level of difficulty, we started the training. The order of the tasks was fixed; that is, the participants always performed the single-matrix task first, followed by the double-matrix task. The average duration of each session was about 22 min (with a range of 11–46 min).

A detailed description of the tasks follows below.

## Single-matrix: memorization of spatial dispositions

**Figure 2.**
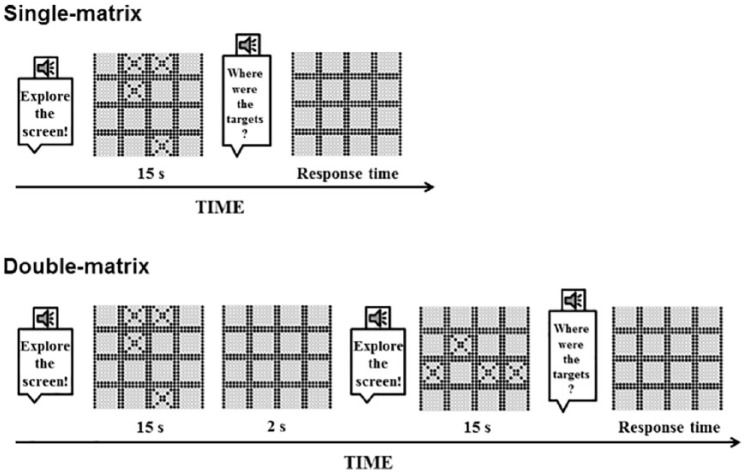
Timeline of a trial. Upper panel: Schematic of successive events within a trial for single-matrix task. A speech synthetized voice indicated to the participant that the targets appeared onscreen. After a presentation time of 15 s, a voice (not reported in the figure) asked the participant to remove the fingers from the display. The targets disappeared and a voice asked the participant to indicate target locations. After each answer, the experimenter started a new trial. Lower panel: Schematic of successive events within a trial for double-matrix tasks. Task events were similar to the Single-matrix task, but two different matrices were displayed in sequence. A 2-s interstimulus interval interleaved matrices presentation. We asked participants to report there were targets in the two matrices by replicating the original temporal sequence (i.e. first matrix first, then second matrix). After each answer, the experimenter started a new trial.

Participants were presented with a single matrix on the Hyperbraille. By “matrix,” we mean a squared grid composed of cells where each cell can be either filled up with a target tactile symbol (a cross, as shown in Figure 1(a)) or not. We implemented four possible matrix sizes: 2 × 2, 3 × 3, 4 × 4 and 5 × 5 (see Figure 2 for an example of 4 × 4 matrix). After the targets disappeared, participants were asked to touch the cells containing the targets. As in Leo et al.,^24^ we manipulated the level of difficulty. In particular, the matrix size and the number of targets were set according to each participant’s ability at the beginning of each session. We ran 10 trials of this task and computed the recall accuracy in percentage (the overall number of correctly recalled targets divided by the number of presented targets). Whenever a participant reached a ceiling effect during a testing session, we increased the level of difficulty. No feedback on performance was communicated to the participants.

## Double-matrix: memorization of spatial dispositions

The double-matrix task was similar to the single-matrix task, but in this case, we presented two different matrices with different target locations in sequence with an interstimulus interval (ISI) of 2 s. The study asked participants separately to recall the target locations by replicating the original temporal sequence (see Figure 2). In other words, they had to report two separate target dispositions. In this instance, all other parameters and procedures (e.g. matrix sizes, timing, level of difficulty estimation, etc.) were the same as in the single-matrix task. As in the case of the single-matrix task, the study afforded no feedback on performance to participants.

## Coding exploration strategies

All sessions of both tasks were videotaped so as to code the manual exploratory strategy used by each participant.

**Figure 3.**
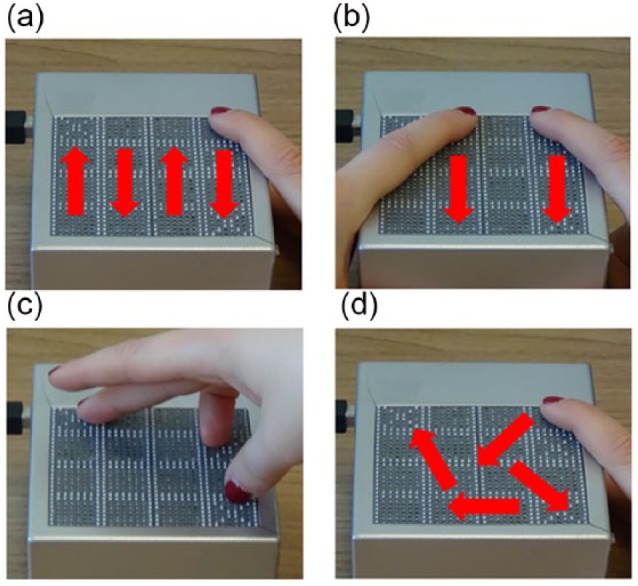
Main exploration strategies used by participants. Panels a, b, c and d show examples of serial, parallel, proprioceptive and random exploration, respectively.

Two experimenters analyzed the videotapes and identified 14 categorical items (see Table 2). Items 1–4 corresponded to four main exploration strategies (see Figure 3): *serial, parallel, proprioceptive* and *random*.

**Table 2.** List of items describing the exploration strategies.

Item #	Name of strategy	Meaning
1	Serial	One or more fingers are used to scan the matrix row by row or column by column
2	Parallel	Two or more fingers are used to scan in parallel two or more rows or columns
3	Proprioceptive	Regardless of the adopted exploration strategy, the participant placed the fingers at the target locations to code a proprioceptive simultaneous memory trace of the target positions
4	Random	Exploration did not follow an apparent exploration strategy (i.e. the participant moved his or her fingers randomly from a cell of the matrix to another).
5	One hand	The participant used one hand to explore the matrix
6	Two hands	The participant used both hands to explore the matrix
7	Time	The exploration lasted less than 15 s
8	One finger	The participant used this number of fingers to explore the matrix
9	Two fingers	
10	Three fingers	
11	Four fingers	
12	Five fingers	
13	Six fingers	
14	Seven fingers	

Each item was scored as “1” or “0” depending on whether that strategy was adopted (e.g. a participant used a “proprioceptive strategy”) or not. Participants received scores for every first and last trial of every one of the four sessions.

Items 5 and 6 corresponded to the use of *one hand* versus *two hands* to explore the tactile stimuli. Item 7 (exploration *time*) indicated whether exploration was performed within the maximum amount of time. The other items (from 8–14) identified the precise *number of fingers* that participants used to explore. No additional items were needed, since the participants never used more than *seven fingers* to perceive the tactile stimuli.

When coding the videotapes, two judges working separately attributed a binary value to each of the 14 items for each participant: “1” if that strategy was adopted, and “0” if otherwise. This was done for the first and the last trial of each session. A pre-screening of the videos showed indeed that participants tended to use the same strategy within a session and sometimes changed it between sessions. The judges considered the items as independent from each other. At the end of the procedure, we computed an interjudgment agreement for each variable (with 73% being the lowest score).

## Statistical analysis

The sample size of visually impaired groups we tested (*n* = 8) is owed to the inherent difficulty in recruiting a large group of early totally blind and low-vision youngsters. However, our sample size is comparable to those in other studies involving visually impaired persons.^17,39–41^

Since data were not normally distributed as verified with Shapiro–Wilk tests, we used non-parametric statistics.

We report for each task (1) performance results: (a) we measured how accuracy varied across sessions (I, II, III and IV) for each group (BLI, LOW, SIG) using Friedman analyses of variance (ANOVAs) followed by Wilcoxon paired tests as post hoc and (b) we measured how performance, for each session, differed across groups using Kruskal–Wallis tests followed by Mann–Whitney tests as post hoc; (2) level of difficulty results: we measured how the matrix size and targets’ number varied across groups both at the beginning and at the end of the training using Kruskal–Wallis tests followed by Mann–Whitney tests as post hoc; (3) exploration strategy results: (a) we ran principal components analysis (PCA) and multidimensional scaling (MDS) analysis to quantify similarity among participants using the coded exploration strategy variables as input, (b) we contrasted groups’ behaviors for each exploration variable (exploration time, number of hands, number of fingers, serial, parallel, proprioceptive, random exploration) using Kruskal–Wallis tests followed by Mann–Whitney tests as post hoc, (c) we computed the correlation between each exploration variable and performance and (d) we investigated whether there were variables predicting a participant’s membership as “blind,” “low-vision,” or “sighted” using discriminant analyses.

We set statistical significance at *p* < .05. Correction for multiple comparisons, whenever necessary, was conducted using the False Discovery Rate (FDR) control based on the Benjamini–Hochberg methods. This has been proven to be a less conservative and more powerful technique than the classical Bonferroni’s correction.^42,43^ Finally, we reported also the following measures of effect size: (1) the Kendall’s *W* concordance for the Friedman ANOVA, (2) the eta-square $\left(\eta^{2}=\chi^{2}/(n-1)\right)$ for the Kruskal–Wallis tests and (3) *r* $\left(r=z/\sqrt{n}\right)$ for Wilcoxon and Mann–Whitney tests. As for the interpretation of the effect sizes, we followed Cohen.^44^ According to his guidelines, for *W* and *r*, a small effect is .1, a medium effect is .3, a large effect is .5. For *η*^2^, a small effect is .01, a medium effect is .06, a large effect is 0.138, a very large effect is .36 and an extremely large effect is .5.

## Results

One BLI performed only one of the two tasks reported in this study (double-matrix task).

As in Leo et al.,^24^ we normalized response accuracy data to the first session baseline. Specifically, this involved accuracies from session II onward, which were converted to percentage performance differences relative to the first session baseline. In this way, we cumulated the relative improvements of the tasks, both when the difficulty levels remained the same across trials and when difficulty levels had to be changed.

## Performance results

### Single-matrix task

Leo et al.^24^ already presented performance results for BLI and LOW in the single-matrix task.

**Figure 4.**
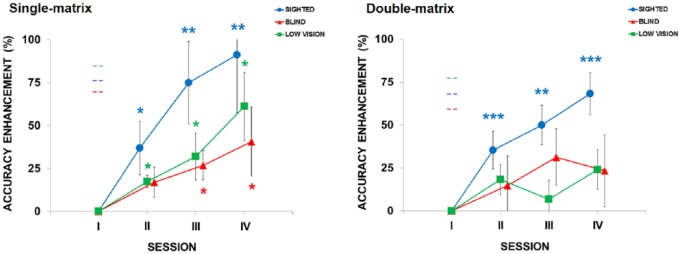
Accuracy results. Left panel: Normalized accuracy enhancement (SEM indicated as whiskers) across sessions in the single-matrix task. Color-coded asterisks indicate a significantly larger accuracy enhancement for that training-session relative to the baseline (*p < .05; **p < .01). Color-coded dashed lines represent the accuracy at the baseline for each group after correcting for the level of difficulty (number of targets). Right panel: Normalized accuracy enhancement (SEM indicated) across sessions in the double-matrix task. Blue asterisks indicate a significantly larger accuracy enhancement relative to the baseline in the sighted group (**p < .01; ***p < .001). Color-coded dashed lines represent the accuracy at the baseline for each group after correction for level of difficulty (number of targets).

The left panel of Figure 4 shows the normalized accuracy enhancement across sessions for BLI, LOW and SIG. All the groups improved their performance during the spatial memory training with the single-matrix task. In fact, the learning effect was statistically significant in the BLI (*χ*^2^ = 12.45; *p* = .006, Kendall’s *W* = .69). The accuracies of sessions III and IV (27% and 41%, respectively) significantly improved compared to the session I baseline (*p*FDR-corrected = .041 and .042, respectively; see Figure 4). Performance improvement was also significant in the LOW (*χ*^2^ = 16.54; *p* = .0009, *W* = .79) and in the SIG (*χ*^2^ = 25.48; *p* = .00001; *W* = .53). All the sessions from the II to the IV in these groups showed significant improvement compared to the baseline (all *p*FDR-corrected < .05).

We then contrasted group performances for each session. Despite a visual trend toward a larger learning effect in the SIG, we observed no statistical difference between groups (all *p*s > .49).

Since the sample numerosity of the BLI and LOW groups was small, we created a unique group of visually impaired youngsters (VIMP) to check whether this lack of statistical differences might be due to the limited sample size. We then compared VIMP and SIG performances for each session. In this case, we observed no significant difference between VIMP and SIG.

### Double-matrix task

The right panel of Figure 4 shows the same analysis for the double-matrix task. The SIG improved significantly during the training (*χ*^2^ = 26.88; *p* = .00001, *W* = .56). Sessions II, III and IV recalling performances (35%, 50% and 68%, respectively) were significantly better than the baseline (all *p*FDR-corrected < .01). On the contrary, the learning effect in the visually impaired groups was weaker. We observed no significant enhancement in the BLI or LOW compared to the baseline.

As with the single-matrix task, we collapsed BLI and LOW into a unique VIMP group. This allowed us to investigate whether the absence of learning effects might be due to the limited sample sizes. Again, we observed no significant learning.

We then contrasted group performances for each session. A significant difference between groups emerged in session IV (*χ*^2^(2) = 6.44; *p* = .039, *η*^2^ = .22). SIG performance is higher than BLI and LOW performance (both *p*FDR-corrected = .069, both *r*s > .41).

In a comparison between VIMP and SIG performances for each session, a difference between groups became evident in session IV (*U* = 46; *p*FDR-corrected = .03, *r* = .47). SIG did better than VIMP.

### Comparison between single- and double-matrix performance

We compared performance improvements of each session in the single- and double-matrix tasks for the VIMP and the SIG groups separately using Wilcoxon paired tests. As for the VIMP, the performance in session IV of the single-matrix task was higher than the corresponding performance of the double-matrix task. This was the case even though this effect does not survive FDR correction (52% vs 25%, *p* uncorrected = .039, *p*FDR-corrected = .11). As for the SIG, task difficulty did not modulate performance.

## Levels of difficulty

### Single-matrix task

Having adapted the initial level of difficulty to each participant’s ability, we also verified whether the three groups differed in terms of levels of difficulty used.

**Figure 5.**
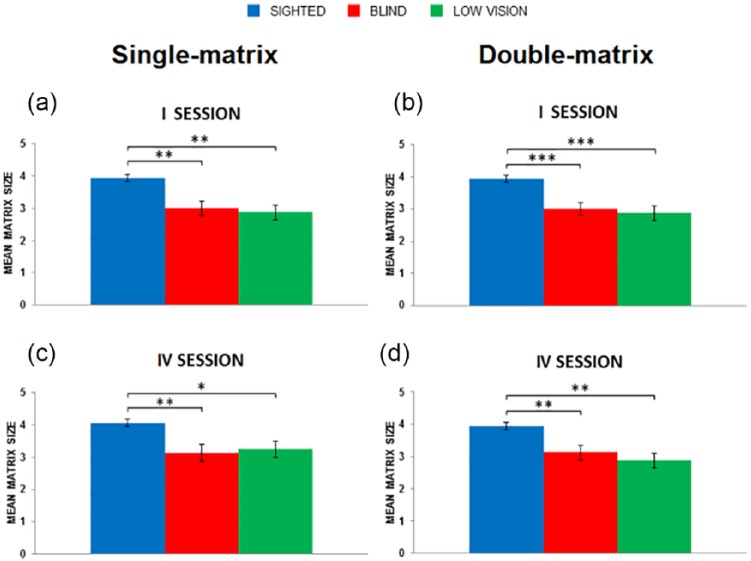
Levels of difficulty used in the single-matrix and in the double-matrix task. The two panels in the left column (a and c) show the average matrix size employed in the single-matrix task in each group at the beginning and end of the training, respectively. The two panels in the right column (b and d) show the average matrix size employed in the double-matrix task in each group at the beginning and end of the training, respectively. Asterisks indicate significant differences between groups (*p < .05; **p < .01; ***p < .001).

Matrix size significantly differed in the three groups, both at the beginning (*χ*^2^(2) = 16.36; *p* = .0003, *η*^2^ *=* *.55*) and at the end of training (*χ*^2^(2) = 12.44; *p* = .002, *η*^2^ = .41). As for session I (see Figure 5(a)), matrix size was significantly larger in the SIG compared to the BLI (3.9 vs 3; *U* = 13.5; *p*FDR-corrected = .0015, *r* = .59) and LOW (3.9 vs 2.9; *U* = 13.5; *p*FDR-corrected = .0012, *r* = .63). On the contrary, matrix size did not differ significantly between BLI and LOW. This trend persisted also in the final session IV (see Figure 5(c)). Matrix size is still larger in the SIG compared to the BLI (4.1 vs 3.1; *U* = 17; *p*FDR-corrected = .0045, *r* = .64) and LOW (4.1 vs 3.3; *U* = 24.5; *p*FDR-corrected = .0045, *r* = .49). We did the same comparisons for the number of presented targets. We observed no statistical differences between groups in the number of targets used either at the beginning or end of training.

### Double-matrix task

In this case, the matrix size differed significantly in the three groups both at the beginning (*χ*^2^(2) = 17.17; *p* = .0002, *η*^2^ = .55) and at the end of training (*χ*^2^(2) = 15.18; *p* = .0005, *η*^2^ = .49). As for session I (see Figure 5(b)), matrix size was significantly larger in the SIG compared to the BLI (3.9 vs 3; *U* = 14.5; *p*FDR-corrected = .00075, *r* = .71) and LOW group (3.9 vs 2.9; *U* = 13.5; *p*FDR-corrected = .00075, *r* = .72). On the contrary, matrix size did not differ significantly between BLI and LOW. This trend persisted into the final session IV (see Figure 5(d)). Matrix size remained larger in the SIG compared to the BLI (3.9 vs 3.1; *U* = 22; *p*FDR-corrected = .003, *r* = .61) and LOW (3.9 vs 2.9; *U* = 13.5; *p*FDR-corrected = .0012, *r* = .71). Again, matrix size did not differ significantly between the BLI and LOW. As for the number of presented targets, the three groups did not differ in sessions I or IV.

## Exploration strategies

As the videos of a single sighted participant were not clear enough, we removed his data from the exploration strategy analyses reported below.

## PCA and MDS

### Single-matrix task

Our first goal was to investigate the relation among the exploration strategies. Since the strategies cannot be assumed as independent, we hypothesized that a PCA could reveal possible similarities between strategies. The input to the PCA consisted of the mean similarity scores between participants based on the 14 exploratory procedure ratings we collected (exploration time, number of hands, number of fingers (from 1 to 7), serial, parallel, proprioceptive or random strategy). We also computed correlation coefficients between each factor and each exploration strategy.

PCA revealed five factors, accounting for 81% of the variance. Factor 1 was positively correlated with using *one* (0.62), *two fingers* (0.84) or the *random strategy* (0.50) and negatively correlated with using *five* (–0.78) and *six fingers* (–0.64). Factor 2 was positively correlated with using *one hand* (0.69) and *six* (0.62) and *seven fingers* (0.58) and negatively correlated with using *two hands* (–0.62) and *four fingers* (–0.61; see Table 3 for a complete description of the five factors).

**Table 3.** PCA results for the single- and double-matrix tasks.

	Factor 1	Factor 2	Factor 3	Factor 4	Factor 5	Factor 6
Single-matrix						
Exploration time	0.11	0.23	**0.71**	0.03	0.07	
One hand	0.45	**0.69**	−0.30	0.18	−0.18	
Two hands	−0.32	**–0.62**	0.44	−0.20	0.12	
One finger	**0.61**	0.10	0.26	−0.40	−0.16	
Two fingers	**0.84**	−0.12	−0.11	−0.01	0.29	
Three fingers	0.03	−0.19	−0.37	**0.80**	0.15	
Four fingers	−0.48	−**0.60**	−0.13	0.33	−0.18	
Five fingers	−**0.78**	0.27	0.11	0.16	−0.30	
Six fingers	−**0.64**	**0.62**	0.35	0.06	0.05	
Seven fingers	−0.48	**0.58**	0.19	0.01	0.09	
Serial	−0.16	0.34	−**0.66**	−0.18	0.41	
Parallel	−0.46	−0.32	−0.15	−**0.63**	−0.04	
Proprioceptive	−0.27	−0.42	0.31	0.27	**0.56**	
Random	**0.50**	−0.02	**0.59**	0.39	−0.32	
Double-matrix						
Exploration time	−0.08	−0.24	0.26	0.10	0.45	**0.73**
One hand	**0.77**	−0.17	−0.05	−0.32	−0.30	0.28
Two hands	−**0.77**	0.03	−0.21	0.18	0.21	−0.35
One finger	**0.68**	−0.04	0.22	−0.22	0.22	−0.04
Two fingers	0.38	0.45	−**0.52**	**0.50**	0.17	−0.02
Three fingers	−0.07	0.38	−**0.57**	0.18	−0.47	0.41
Four fingers	−**0.62**	0.24	−0.09	−0.39	−0.31	0.20
Five fingers	−0.42	−**0.71**	−0.14	−0.12	−0.39	−0.05
Six fingers	−0.26	−**0.88**	−0.07	0.16	−0.17	0.005
Seven fingers	−0.19	−**0.52**	0.42	**0.56**	−0.01	0.13
Serial	0.38	0.20	0.36	**0.55**	−0.48	−0.13
Parallel	−0.48	0.40	0.43	−0.29	−0.01	−0.08
Proprioceptive	−**0.69**	0.34	−0.09	0.15	0.15	0.29
Random	0.21	−**0.53**	−**0.72**	−0.10	0.29	−0.07

The factors with eigenvalues >1 are reported. The numbers represent correlation between exploration strategies and factors. Numbers in bold indicate stronger correlations (>.5). PCA: principal components analysis.

**Figure 6.**
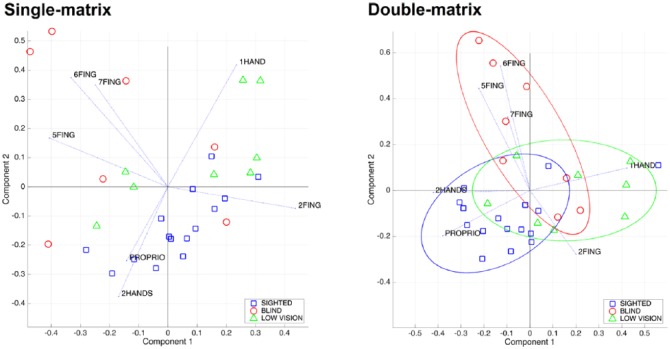
Multidimensional scaling analysis. Two-dimensional scaling solution for participants based on exploratory procedure ratings of single-matrix task (left panel) and double-matrix task (right panel). Strategies are shown as vectors in the two main components plane. Although we used all 14 items, here we depict only the most relevant strategies that account for most of the variance in the two-dimensional (2D) space. The diagrams depict blind participants as red circles, low-vision as green triangles and sighted as blue squares. The right panel colored ellipses show how the three tested populations of youngsters concentrate in different areas of the 2D space as defined by the two components. Note that sighted youngsters shared a larger use of *two hands* and the *proprioceptive strategy*, whereas low-vision youngsters typically used only *one hand* and blind youngsters used multiple fingers to explore.

Then, we ran a MDS to quantify similarity among participants, that is, to represent participants and their strategies in a unique graphical space. The MDS allows to represent the variables that in the PCA were positively correlated with a factor close to each other and in opposite direction than the variables that were negatively correlated. Another goal of the MDS was to identify behavioral similarities among participants or, on the contrary, any strategies exclusively adopted by specific participants and possibly mediated by the level of visual disability. Participants who were more similar are closer together on the graph than those who are dissimilar. We used a standard nonmetric Guttman–Lingoes as starting configuration. We decided for a two-dimensional solution because it reflects a good compromise between map readability and low stress value (0.11). Although there are no objective criteria to establish a threshold for acceptable stress values, Monte Carlo studies demonstrated that stress values under 0.2 indicate a good fit between output configuration and similarity data.^45^ Figure 6 displays the resulting map.

Qualitatively, a clear clusterization of the three groups of participants is not evident suggesting that the exploration strategies are not consistently modulated by the degree of visual ability in the single-matrix task.

### Double-matrix task

PCA revealed six factors, accounting for 84% of the variance. Factor 1 was positively correlated with the use of *one hand* (0.77) and *one finger* (0.68) and negatively correlated with the use of *two hands* (–0.77), *four fingers* (–0.62) and a *proprioceptive strategy* (–0.69). Factor 2 is negatively correlated with the use of at least *five fingers* as well as with using a *random strategy* (see also Table 3).

As for the MDS, also in this case, a two-dimensional solution allowed to obtain a good fit (stress value = 0.12). In this task, SIG tended to form a dense and separate cluster along lower scores of component 1 (see Figure 6). On the contrary, visually impaired groups were more interspersed along higher scores of component 1.

## Group-wise occurrence of exploration strategies

### Single-matrix task

We performed Kruskal–Wallis ANOVAs for each variable in the MDS analysis.

**Figure 7.**
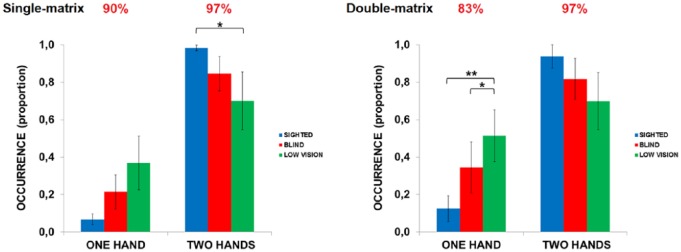
Number of hands used. Proportion of occurrence of *one hand* only exploration and *two hand* exploration in the single-matrix (left panel) and double-matrix tasks (right panel). Red numbers above the histograms indicate the average interjudgment agreement for that variable. Asterisks indicate significant differences between groups (*P < .05; **P < .01).

First, it became evident that the three groups did not differ in terms of *exploration time*. On the contrary, they differed in terms of the number of hands they used in exploration (see Figure 7). We observed a significant effect of the variable *one hand* (*χ*^2^(2) = 6.06; *p* = .048, *η*^2^ = .21). Particularly, there was a trend toward using only *one hand* in the LOW compared to the SIG (*U* = 27; *p*FDR-corrected = .052, *r* = .44). The groups differed also in their use of *two hands* (*χ*^2^(2) = 7.86; *p* = .0196, *η*^2^ = .27). SIG tended to use *two hands* more than BLI (pFDR-corrected = .06, *r* = .29) and significantly more than LOW (pFDR-corrected = .017, *r* = .44).

**Figure 8.**
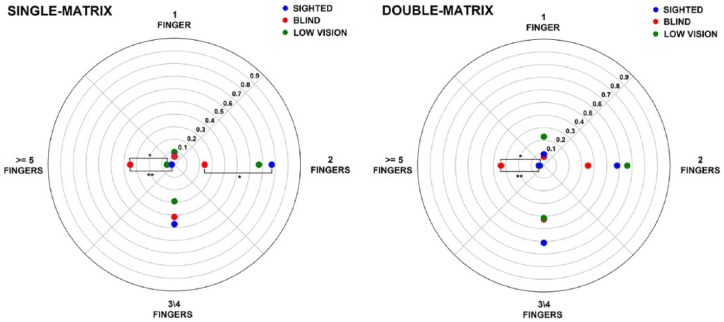
Number of fingers used. Proportion of use of different number of fingers in the single-matrix (left panel) and in the double-matrix task (right panel). Asterisks indicate significant differences between groups (*p < .05; **p < .01).

The degree of visual disability also influenced the number of fingers participants used to explore. BLI used more fingers when exploring than the LOW (*p*FDR-corrected = .025) and SIG (*p*FDR-corrected = .006, see also Figure 8). On the contrary, SIG more often used only *two fingers* compared to the BLI (*p*FDR-corrected = .023).

**Figure 9.**
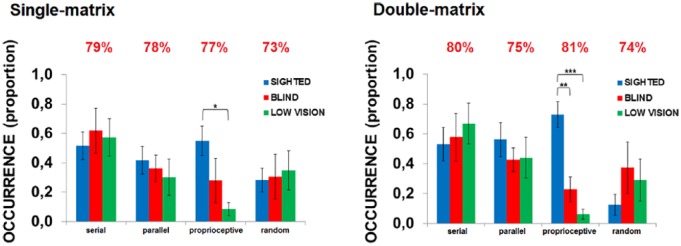
Exploration strategies. Proportion of use of different exploration strategies in the single-matrix (left panel) and double-matrix task (right panel). Red numbers above the histograms indicate the average interjudgment agreement for that variable. Asterisks indicate significant differences between sighted and visually impaired youngsters when using the proprioceptive strategy (*p < .05; **p < .01; ***p < .001).

Finally, we investigated whether the three groups differed in the exploration strategy they employed. Groups’ haptic behavior looked very similar regarding the *serial*, the *parallel* and the *random exploration*. On the contrary, we observed a trend toward group differences for the *proprioceptive strategy* (*χ*^2^(2) = 5.98; *p* = .05, *η*^2^ = .21). This is mainly because SIG more often used a *proprioceptive strategy* compared to LOW (*U* = 24; *p*FDR-corrected = .048, *r* = .51; see Figure 9).

### Double-matrix task

The degree of visual disability affected the number of hands used to explore (*χ*^2^(2) = 9.01; *p* = .01, *η*^2^ = .29). LOW more often used only *one hand* to explore than SIG (*p*FDR-corrected = .0.012; see Figure 7). BLI also tended to more often use only *one hand* than SIG did (*p*FDR-corrected = .064).

Moreover, in the double-matrix task, we observed no group differences in terms of *exploration time*. We observed group differences in the number of fingers used for exploring. BLI used more multiple fingers than LOW and SIG (*p*FDR-corrected = .04 and .009, respectively, see also Figure 8).

Finally, we investigated whether the three groups differed regarding the exploration strategy participants used. As with the single-matrix task, visual ability did not modulate the *serial*, the *parallel* and the *random* strategies. On the contrary, we observed a significant group effect for executing the *proprioceptive* strategy (*χ*^2^(2) = 17.18; *p* = .0002, *η*^2^ = .55). SIG used this strategy significantly more than BLI and LOW (both *p*FDR-corrected < .01; see Figure 9).

## Correlation between exploration variables and accuracy

### Single-matrix task

We investigated how the final accuracy enhancement correlates with each exploration variable. To do so, we computed Spearman’s rank correlation coefficients with consideration to the whole sample of participants. We could not find any significant correlation between exploration strategies and final level of performance in the single-matrix task.

### Double-matrix task

None of the correlations was significant after FDR correction. Nevertheless, using only *one hand* is negatively correlated with accuracy (*r_s_* = –.37, *p* uncorrected = .041, *p*FDR-corrected = .24). Using the *proprioceptive strategy* instead tends to be positively correlated with accuracy enhancement (*r_s_* = .42, *p* uncorrected = .018, *p*FDR-corrected = .24). Interestingly, we observed that blind youngsters who more often employed a *proprioceptive strategy* (proportion of use > 0.2) saw larger improvement in session IV compared to their counterparts who did not use it (34% vs 14%), suggesting that also blind youngsters can proficiently and spontaneously use this strategy. All the other correlation coefficients are well above the significance level.

## Discriminant analysis

### Single-matrix task

In an attempt to determine a set of variables that might predict a classification of individuals as “blind,” “low-vision” or “sighted,” all the considered exploration variables were entered in a discriminant analysis. In the results, none of the variables discriminated significantly between the three groups.

### Double-matrix task

Here, we found a variable that discriminated significantly between groups, *F*(28, 32) = 2.10, *p* = .02, *η*^2^ = .88, that is, the *proprioceptive strategy* (*p* < .05). The discriminant function, including all parameters, produced a classification accuracy of 84.4%. Classification accuracy for BLI was 62.5%. We only classified three out of eight BLI as low vision. On the contrary, classification accuracy for LOW and SIG was rather high (100% and 87.5%, respectively).

## Discussion

In the present work we showed that

1. All the youngsters significantly improved their performance in the easier (single-matrix) spatial memory task, regardless of the degree of visual ability, whereas when the cognitive load of the task is higher (double-matrix), sighted youngsters showed learning effects while enhancement in the visually impaired youngsters was reduced;
2. Groups with different visual ability tended to differ in terms of the exploration strategies they employed;
3. There has emerged an ideal exploration strategy that could be exploited in rehabilitation programs.

### Low cognitive load facilitates memory enhancement

As for the first point, a previous study^24^ showed evidence of the possibility of observing learning effects in a simple spatial memory task in blind youngsters. Similarly, learning effects following spatial training have been observed in blind adults performing navigation or shape recognition tasks^21,46–49^ and the level of expertise with raised line materials is associated with higher performance in various spatial tasks.^50,51^ However, the literature highlighted peculiar difficulties in blind adults when two separate spatial configurations must be simultaneously maintained in memory.^15^ As a consequence, a crucial applied research question pertains to whether this skill is adequately trainable in visually impaired youngsters. To expand and answer this question, we implemented training that involves four sessions with a weekly schedule in which blind and blindfolded low-vision and sighted youngsters had to perform two spatial memory tasks that varied in complexity. In the first, simpler task, youngsters had to recall the location of the targets presented in a single matrix, as in Lea et al.^24^ In the second, more demanding task, they had to serially recall the target locations consecutively presented in two separate matrices.

Our results showed robust learning effects in all groups in the single-matrix task. The cumulative level of performance improvement compared to session I was around 41% in the blind group, 61% in the low-vision and 91% in the sighted group. While at a descriptive level, there was a clear trend toward greater performance enhancement in participants with higher visual ability, we could not observe significant group differences relative to performance enhancement. At the same time, sighted youngsters started training with a higher level of difficulty compared to visually impaired groups; this is expressed by the more complex matrices used for testing. Certainly, this introduces a difference in testing conditions between groups, but if we had used the same level of difficulty, the experiment would likely have been affected by ceiling and/or floor effects in performance. Despite different levels of difficulty, our results suggest that SWM in visually impaired youngsters is trainable, at least in tasks characterized by a low cognitive load. Furthermore, the training we implemented using a programmable tactile display could be carried out without the presence of a rehabilitation practitioner, thereby favoring the autonomy of blind people in spatial learning tasks.

As for the double-matrix task, we instead observed a different performance in youngsters with different degrees of visual impairment. While the final level of performance in the sighted group is higher than the baseline, enhancement in the two visually impaired groups is lower and statistically indifferent from the initial level. Importantly, the learning effect is absent when one considers the two visually impaired groups as a single group. Furthermore, performance enhancement in the sighted was higher compared to the visually impaired in session IV of the training. This finding corroborates Vecchi et al.’s^15^ results showing difficulties in blind adults when recalling separate spatial configurations.^51^ Furthermore, as in the single-matrix task, sighted controls started the training with a higher matrix complexity. This result is in agreement with studies showing performance differences in spatial memory tasks between blind and sighted adults^52^ and children.^53^ For instance, Cornoldi et al.^54^ noted difficulties for blind people compared to sighted when using three-dimensional matrices to code imaginary pathways. Similarly, Millar^53^ demonstrated larger errors in blind children compared to sighted in recalling landmark positions on a spatial map.

Differences in performance between blind and sighted people do not always flow in the direction of a better performance among the latter. A recent study comparing working memory in blind versus sighted children found better performance in the former group, not only in short-term memory tasks as already found in other studies,^55–57^ but also in working memory tasks—specifically verbal working memory tasks.^58–61^ Similarly, Raz et al.^62^ reported better verbal serial memory in blind adults than in sighted control counterparts. According to some authors, such an advantage for blind participants would be limited to tasks involving the phonological loop; it would not apply to tasks involving the executive system, such as in our experiments.^57^

### A proprioceptive strategy subtending higher visual capabilities

Regarding the second point, we first analyzed the exploration modalities on a single-subject basis. Particularly, for each trial of both tasks we coded whether participants completed the exploration before the time limit, how many *hands* and *fingers* they used to explore and which haptic strategy (*serial, parallel, proprioceptive, random*) they used most prevalently. As expected, group differences emerged. First, unexpectedly, visually impaired participants used more only *one hand* compared to sighted controls, in particular in the double-matrix task. Previous studies contradicted this, in that blind users tend to spontaneously use *two hands* when exploring (e.g. 31,33,34). This difference might be due to the nature of the tactile patterns employed in the different studies. While we used structured small-size matrices, in Rovira’s et al.^31^ and Perkins and Gardiner^34^ studies, the tactile patterns were more complex (e.g. geometrical shapes or tactile maps of environments) and the material was at least A4 size. In our study, we showed that our blind youngsters used much more frequently multiple fingers (five or more) during exploration than the other groups. In the single-matrix task, the mode of the distribution of number of fingers used by the blind participants is 5, whereas the mode is 2 in the low-vision and sighted groups. As for the double-matrix task, the pattern is similar, but sighted youngsters tended to use more fingers in this than in the single-matrix task. Our results show that even though blind youngsters often use *one hand*, they used the *whole* hand, as reported in Davidson and colleagues.^63,64^ As for exploration strategies, sighted youngsters used significantly more the *proprioceptive* modality compared to visually impaired groups. This difference has been especially evident in the double-matrix task, which is when the task’s imposed cognitive load is higher. Furthermore, a discriminate analysis showed that the *proprioceptive strategy* in the double-matrix task is the only exploration variable that significantly discriminates between groups.

### “Use proprioception and two hands, please”

To identify a possible ideal exploration strategy, we correlated the exploration strategies with performance. For the single-matrix task, we could not find any variable that significantly correlated with performance. Similarly, a discriminate analysis indicated that none of the exploration modalities discriminates between groups. We identified no group-related differences in terms of performance enhancement, even though we observed a trend toward a larger learning effect in the sighted group. Instead, when the SWM load increased, some winning strategies started to emerge. Particularly, performance enhancement was negatively correlated with using only *one hand* and directly correlated with the *proprioceptive strategy*. This exploration modality, as previously discussed, also significantly discriminates between groups. In fact, sighted youngsters, who showed better performance in the double-matrix task, used it significantly more than both the blind and low-vision groups. Visually impaired youngsters might obtain worse results because they use more the *serial* and *parallel strategies*, which typically require a bigger cognitive load since they need a continuous information update. The *proprioceptive strategy* instead allows a global and simultaneous representation of tactile information since it focalizes only on points of interest, that is, targets, reducing the required cognitive load.^65^ This strategy is an example of chunking, which is a cognitive process by which several pieces of information are bound together into a meaningful and coherent whole, to facilitate memorization of items.^66^

One might wonder why sighted participants adopt this winning strategy more often than the blind and low-vision ones. We speculate that this result is explainable by the privileged ways in which blind and sighted acquire information. Blind persons use touch and audition, which are mainly sequential, to encode information, while sighted use mostly vision, which allows holistic acquisition of information.

### Practical implications for rehabilitation

The fact that using two hands and a proprioceptive strategy is correlated with better performance suggests the intriguing possibility that a rehabilitative treatment focused on the augmentation of these two strategies might boost the SWM of visually impaired youngsters. We indeed observed that blind youngsters who more often used the *proprioceptive strategy* had a larger improvement in session IV compared to their counterparts who did not (34% vs 14%). The fact that some blind youngsters are able to use spontaneously the proprioceptive strategy is very important also because it indicates that there may be common strategies between blind and sighted youngsters, as reported also by other authors.^41^ Training one’s ability to keep different spatial information in memory simultaneously can be especially relevant in learning disciplines, such as mathematics and geometry. Such disciplines constitute the theoretical basis of orientation and mobility skills, which require learning and memorizing maps and the spatial dispositions of different objects. The efficient development of working memory skills is especially relevant because these abilities are involved in many academic and intellectual functions, ranging from reading to math, geometry, listening comprehension, fluid reasoning and complex learning.^67^ With this in mind, working memory span might predict learning and career outcomes.^68–70^

### Limitations

The sample size of the visually impaired groups is small in our study. As a consequence, it might be possible that some differences between groups would become significant with larger sample sizes. In the attempt to overcome this limit, we performed some analyses in which we merged the blind and low-vision groups. Future studies might add reliability to our findings, especially when testing larger sample sizes also to statistically validate the observation that blind youngsters using the proprioceptive strategy actually obtain higher performances.

This study did not implement a pre–post test design, so we cannot conclude that the learning effects we observed are generalizable to different working memory tests or even to other spatial skills. Finally, the size of our programmable tactile display might affect exploration strategies. For instance, we showed that blind youngsters often use only one hand when exploring, which is a haptic behavior that might be due to the small size of the tactile display. Future studies might want to investigate how exploration strategies vary as a function of the size of programmable tactile displays.

### Implications of results

One of the implications of this work that one should not underestimate is our use of a programmable tactile display for task implementation. This technological solution allowed the study to overcome several limitations of current rehabilitation methods (e.g. swell paper). First, tactile information can be presented dynamically; second, the tactile content can be adapted more easily to the needs of the single user, for instance, by manipulating the level of difficulty of the task. Finally, such technology can favor (at least in principle) larger autonomy among blind users thanks to the feedback devices can provide.

## Conclusion

This study showed that SWM is trainable in blind, low-vision and sighted youngsters through tactile stimulation, although learning effects are modulated by task complexity and visual disability. From a practical rehabilitation viewpoint, we highlight two main findings: (1) programmable tactile displays can be an aid tool in self-rehabilitation of visually impaired youngsters and (2) spontaneous optimal tactile exploration emerges that might be exploited within rehabilitation contexts.
